# The cardiovascular-immune axis: crosstalk and therapy in atherosclerosis, myocarditis and vasculitis

**DOI:** 10.3389/fimmu.2026.1781248

**Published:** 2026-04-07

**Authors:** Yuan-peng Liao, Yu-xin Wei, Feng-mei Zhang, Zhao-shan Zhang, Sen-ping Xu, Yong-hao You, Jia-wei Guo

**Affiliations:** 1 Department of Cardiovascular Surgery, The First Affiliated Hospital of Yangtze University, Jingzhou, Hubei, China; 2 Department of Pharmacology, School of Medicine, Yangtze University, Jingzhou, China

**Keywords:** atherosclerosis, cardiovascular disease, immune-cardiovascular axis, myocarditis, vasculitis

## Abstract

Atherosclerosis (AS), myocarditis and vasculitis constitute a spectrum of prevalent cardiovascular diseases (CVDs) where immune dysregulation acts as a central pathogenic driver. Consequently, targeting the immune-cardiovascular axis represents a promising therapeutic frontier. This review systematically elucidates the shared immunological mechanisms underpinning these distinct yet interconnected conditions. The specific pathogenic landscapes are dissected, ranging from lipid-driven endothelial dysfunction and plaque instability in AS, to pathogen- or autoimmune-mediated myocardial injury in myocarditis, and necrotizing vessel wall inflammation in vasculitis. The fundamental roles of innate and adaptive immunity in driving cardiovascular pathology are delineated, highlighting the significant cross-talk and convergent immunological signatures among AS, myocarditis and vasculitis. Central to this convergence, CXCR4, PYCARD, TSC22D3 (GILZ), and HSPA1A are identified as critical hubs orchestrating leukocyte trafficking, inflammasome activation, immune tolerance, and proteostatic stress, respectively. Furthermore, precision strategies targeting these hubs are evaluated, utilizing agents such as Plerixafor, Lycorine, Dexamethasone, and Tanespimycin. Finally, emerging frontiers, including natural products and biomaterials, are assessed, providing a perspective on current clinical trials and future directions for resolving cardiovascular inflammation.

## 1 Introduction

Cardiovascular diseases (CVDs) remain the preeminent cause of global morbidity and mortality. While historically characterized by hemodynamic or metabolic disturbances, accumulating evidence underscores that CVDs pathogenesis is fundamentally orchestrated by the aberrant activation of the cardiovascular-immune axis. The cardiovascular system relies on a sophisticated homeostatic interplay to maintain energy supply and metabolic exchange. Crucially, its structural stability depends not only on the integrity of vascular and myocardial cells but is intrinsically governed by immune surveillance (1).

The immune system maintains a dichotomous role in cardiovascular physiology: it serves as both a vital regulator of homeostasis and a potent driver of pathology (2). This “Janus-faced” nature implies that while a calibrated immune response is prerequisite for tissue repair and remodeling following acute or chronic injury, an unchecked or imbalanced inflammatory cascade precipitates maladaptive remodeling and accelerates disease progression. Thus, the distinction between physiological repair and pathological damage is determined by the magnitude and duration of the immune response (1).

In recent years, AS, myocarditis and vasculitis have emerged as distinct yet mechanistically overlapping paradigms of cardiovascular inflammation. Despite anatomical differences, these conditions exhibit significant pathogenic cross-talk rooted in shared immunological dysregulation. This review aims to decipher the convergent mechanisms underpinning these three prototypical CVDs, emphasizing their reciprocal remodeling and molecular convergence. Through a comprehensive synthesis of the cardiovascular-immune landscape, critical molecular hubs are identified, providing a theoretical scaffold for the development of precision immunotherapeutic strategies.

## 2 Innate and adaptive immunity

The cardiovascular system operates under the vigilant surveillance of two distinct yet synergistic immunological arms: innate and adaptive immunity. These systems function not merely as defensive barriers against pathogens but as integral regulators of cardiovascular homeostasis, facilitating tissue repair and metabolic balance. However, in pathological contexts such as AS, myocarditis and vasculitis, the dysregulation of these immune responses transforms them from protective guardians into drivers of chronic inflammation and tissue injury.

The innate immune system serves as the first line of defense, comprising a diverse repertoire of cellular effectors, including monocytes, neutrophils, macrophages, dendritic cells (DCs), natural killer (NK) cells, and mast cells (MCs) (3), alongside humoral components such as cytokines, chemokines, and the complement system (4). Central to its function is the capacity to rapidly detect danger signals through pattern recognition receptors (PRRs). Upon recognition of pathogen-associated molecular patterns (PAMPs) or damage-associated molecular patterns (DAMPs) released during cardiovascular injury, innate immune cells initiate an immediate inflammatory cascade. This activation triggers the secretion of chemokines to recruit circulating leukocytes to the site of injury, establishing a pro-inflammatory microenvironment characterized by the release of key cytokines such as TNF-α, IL-1β, and IL-6 (2, 4, 5). While this response is evolutionarily designed to contain infection and initiate repair, its persistent activation in CVDs fuels a maladaptive cycle of chronic inflammation and tissue remodeling (6).

Complementing the rapid, non-specific nature of innate immunity, the adaptive immune system provides a highly specific and enduring response essential for distinguishing self from non-self antigens (7). This system is orchestrated principally by B lymphocytes and T lymphocytes, which utilize unique antigen receptors, specifically B-cell receptors (BCRs) and T-cell receptors, to recognize specific antigenic determinants (4, 7). The activation of adaptive immunity is intimately coupled with the innate system, relying on antigen presentation by professional antigen-presenting cells (APCs) (8). Following antigen engagement, naive CD4+ T cells differentiate into distinct effector lineages, including T helper cells and regulatory T cells (Tregs), depending on the local cytokine milieu (6). Functionally, CD4+ T helper cells coordinate the immune response by modulating other leukocyte populations, whereas CD8+ cytotoxic T cells directly execute the apoptosis of virus-infected or malignantly transformed cells (9). This sophisticated cellular network works in concert with innate effectors to maintain cardiovascular integrity, yet its aberrant activation against self-antigens can precipitate autoimmune-mediated cardiovascular damage (2).

Beyond classical antigen recognition, immune regulation integrates sophisticated metabolic and neural signaling. Mechanistically, vascular macrophages expressing Olfactory Receptor 2 (OLFR2) sense the lipid peroxidation byproduct octanal (10, 11). Octanal binding triggers calcium influx to activate the NLRP3 inflammasome and IL-1β secretion, linking lipid dysregulation to vascular inflammation (10, 11). Concurrently, the neuro-immune axis modulates inflammation via the vagal anti-inflammatory reflex and macrophage α7 nicotinic acetylcholine receptors (α7nAChR) (12, 13). This signaling shifts T cell differentiation by suppressing Th1/Th17 responses and promoting Tregs (12, 13).

While immune responses maintain cardiovascular homeostasis physiologically, their disruption via metabolic or neural dysregulation drives chronic inflammation in pathologies such as AS, myocarditis and vasculitis. Thus, targeting these immunomodulatory pathways offers a promising therapeutic strategy.

## 3 Atherosclerosis

### 3.1 Background

Atherosclerosis (AS) remains the preeminent cause of cardiovascular morbidity and mortality globally (14). It is fundamentally recognized as a chronic inflammatory disease that serves as the pathological basis for numerous cardiovascular and metabolic disorders (15–17). The disease is characterized by the accumulation of lipids, inflammatory cells, and immune effector cells within the arterial wall, triggering a cascade of endothelial injury, lipid deposition, macrophage activation, oxidative modification, and smooth muscle cell (SMC) migration. These processes ultimately culminate in plaque formation and vascular wall thickening (18–20).

Structurally, AS is defined as a vascular pathology occurring within the intima (21, 22). The plaque microenvironment is a heterogeneous ecosystem comprising endothelial cells (ECs), leukocytes, foam cells, modified lipids, and inflamed SMCs (23), often initiating as lipid streaks (18). This complex milieu involves multiple cell types, including T cells, B cells, macrophages, and MCs, which orchestrate the local immune response (16, 24).

The pathogenesis of AS is multifactorial, involving lipid metabolism disorders, oxidative stress, endothelial dysfunction, foam cell formation, SMC migration, inflammation, and extensive cell death (25, 26). Lipid metabolism disorders (7), particularly the subendothelial retention of low-density lipoprotein (LDL) (27, 28), act as a primary trigger. In the subendothelial space, LDL undergoes oxidative modification to form oxidized LDL (ox-LDL). This recruits monocytes, which differentiate into macrophages and generate excessive reactive oxygen species (ROS) (14, 29), driving disease progression via oxidative stress pathways.

Ultimately, the progression of unstable plaques, driven by foam cell formation and the accumulation of cholesterol and cellular debris, leads to the formation of a fibrous cap. Under conditions of hemodynamic shear stress (30), this pathological intimal thickening can result in partial or complete blood flow obstruction (31), precipitating major adverse cardiovascular events such as myocardial infarction and stroke (23, 32). Thus, through the interplay of these etiological mechanisms, AS develops progressively, establishing itself as the primary causative factor in acute and chronic CVDs.

#### 3.1.1 Immunological mechanisms of atherosclerosis

**Figure 1:**
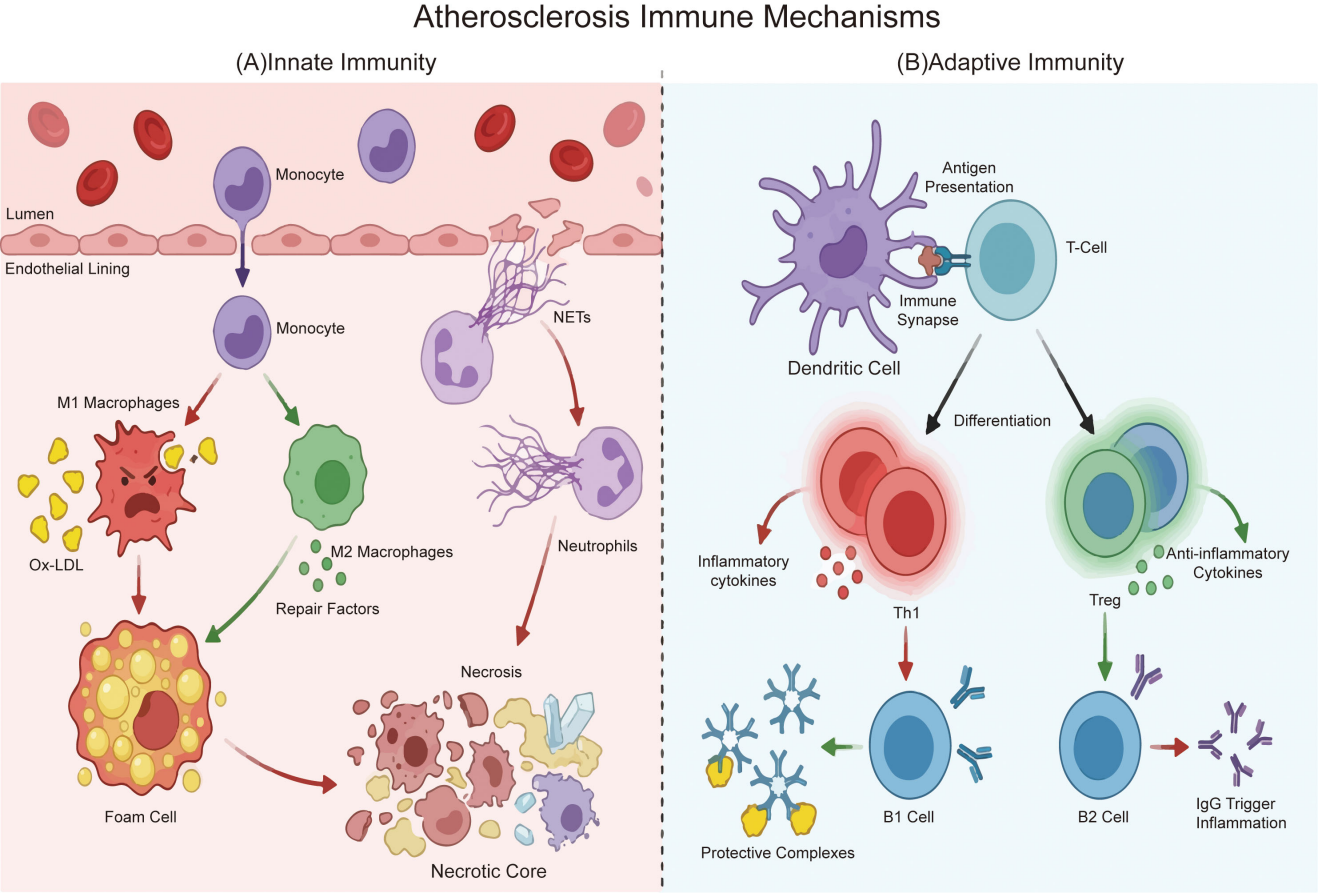
Schematic representation of innate and adaptive immune responses in atherosclerosis. The diagram illustrates the functional dichotomy between innate and adaptiveimmunity within the arterial intima. **(A)** Innate immunity. Circulating monocytes are recruited across the endothelium and differentiate into macrophages. Pro-inflammatory M1 macrophages engulf oxidized LDL (ox-LDL) particles to form foam cells, contributing to the necrotic core at the base. Neutrophils release web-like neutrophil extracellular traps (NETs) to exacerbate inflammation. In contrast, M2 macrophages exhibit reparative phenotypes. **(B)** Adaptive immunity. Dendritic cells (DCs) form immune synapses to present antigens to naive T cells. Subsequent T cell differentiation leads to either pro-inflammatory Th1 cells or atheroprotective regulatory T cells (Tregs). B cell subsets also diverge, with B1 cells secreting natural IgM antibodies and B2 cells producing IgG. Red and green arrows denote pro-inflammatory and anti-inflammatory signaling pathways, respectively. ox-LDL, Oxidized Low-density lipoprotein; NETs, Neutrophil extracellular traps; DCs, Dendritic cells; Tregs, Regulatory T cells.

AS represents a prototypical chronic inflammatory disease in which both innate and adaptive immunity play orchestrating and complex roles (33, 34). The immune response involves a network of key cell subpopulations (**Figure 1**), including DCs, MCs, macrophages, T cells, and B cells (4, 35, 36), as well as interactions between lymphocytes and monocytes (15). Crucially, immune responses mediated by these cells can impair endothelial function, which in turn amplifies the immune cascade.

### 3.2 Innate immune cells

In the context of AS, innate immune cells act as the primary effectors of the immune response. Representative participants include macrophages, monocytes, neutrophils, DCs, NK cells, and MCs (2, 37).

#### 3.2.1 Macrophages

Macrophages dominate atherosclerotic plaques (17), exhibiting a dynamic phenotype that evolves from protective scavenging to pro-inflammatory destruction (25, 38). Initially, resident macrophages maintain homeostasis via efferocytosis (39, 40). Beyond traditional CD36-mediated uptake, Cdc42/Rac1-driven macropinocytosis significantly contributes to LDL accumulation and foam cell formation (41). Pathologically, excessive ox-LDL uptake facilitates foam cell entrapment (31, 42), leading to necrosis and necrotic core expansion (6, 43). Mechanistically, cholesterol crystals and ox-LDL trigger NLRP3 inflammasome activation, while simultaneously amplifying inflammation through antigen presentation (44, 45).

Functionally, macrophages differentiate into pro-inflammatory M1 or reparative M2 phenotypes (46, 47), alongside distinct subsets like TREM-2hi (15). M1 macrophages release ROS and cytokines including IFN-γ and TNF-α to intensify vascular remodeling (1, 3, 17), whereas M2 subtypes secrete IL-10, TGF-β, and IL-38 to mediate repair (3, 6, 48). However, endothelial ROS can skew polarization toward the M1 phenotype via NF-κB signaling (48, 49). Crucially, metabolic interventions like lactate-mediated MeCP2 modification at K271 can restore M2 dominance (50). This balance dictates calcification patterns, with M1 promoting destabilizing microcalcifications versus M2-associated stabilizing macrocalcifications (51).

#### 3.2.2 Monocytes

Monocytes are key innate immune cells that drive plaque growth. They bind to adhesion molecules expressed by activated ECs and are recruited to the intima via chemokines. Once infiltrated, monocytes mature into macrophages expressing scavenger receptors, phagocytose lipoproteins, and differentiate into foam cells (21, 52). Throughout the progression of AS, monocytes play a pivotal role in plaque formation, stabilization, and rupture (53). Senescent or necrotic ECs increase the secretion of chemokines such as Monocyte Chemoattractant Protein-1 (MCP-1), thereby recruiting additional peripheral monocytes. This accelerates foam cell formation and drives rapid plaque progression (31).

Functionally, monocytes contribute to phagocytosis, antigen presentation, and inflammation (3). Within the plaque, monocytes differentiating into macrophages release cytokines and growth factors upon necrosis, further exacerbating inflammation (29). This response enhances DAMPs, which, together with mitochondrial ROS, accelerate cell death (48), thereby promoting the expansion of the atherosclerotic necrotic core.

Beyond macrophage differentiation, monocytes can also differentiate into DCs (3), facilitating cytokine signaling, antigen presentation, and the regulation of T cell responses (26). Equipped with chemokine receptors and PRRs, monocytes are specialized circulating cells capable of rapid pathogen recognition and phagocytosis (44). In summary, by differentiating into macrophages and DCs, monocytes play multiple critical roles in the initiation, maintenance, and destabilization of atherosclerotic plaques.

#### 3.2.3 Neutrophils

Recruited early by hemodynamic disturbances or DAMPs, neutrophils initiate endothelial damage and monocyte recruitment via oxidative and proteolytic mediators, including ROS, elastase, and MMP-8/9 (1, 2, 32, 52). A critical pathogenic mechanism involves Neutrophil Extracellular Traps (NETs), which damage ECs (54) and activate the macrophage NLRP3 inflammasome to release IL-1β and IL-18, thereby perpetuating plaque instability and thrombosis (55–57). Additionally, neutrophil-derived EVs deliver miR-155 to activate endothelial NF-κB signaling (18). However, neutrophils exhibit plasticity (46). Reparative N2 neutrophils facilitate resolution via Lipocalin-2 and S100A8/A9 (56) and mediate angiogenesis through VEGF-A/VEGF-D (46, 58, 59). Yet, VEGF-A-driven neovascularization is often immature, paradoxically increasing hemorrhage risk (60). Given that neutrophil depletion can exacerbate fibrosis (1), therapeutic strategies must precisely target their activation status.

#### 3.2.4 Dendritic cells

DCs bridge innate and adaptive immunity, accumulating in atheroprone regions (15). They phagocytose ox-LDL via receptors such as SR-A, CD36, and LOX-1 (8) to present self-antigens including ApoB100 and HSP60 to T cells (8, 39). Analogous mechanisms drive myocarditis, where DCs activate autoreactive T cells via α-myosin heavy chain (35, 61), while simultaneously governing post-injury repair through apoptotic cell clearance and macrophage regulation (2). While ox-LDL exerts complex effects on maturation (62), mature DCs expressing CD40 and CD86 activate naive T cells via cytokines including IL-12, IL-23, and IFN-α/β (31, 34). Functionally, DC roles are subset-dependent: CD11b+ and CCL17+ cDCs are generally pro-atherogenic, whereas Flt3-dependent CD103+ cDCs induce protective Treg responses (15, 37). Consequently, DCs regulate T cell differentiation and plaque stability (39, 63). Since immature DCs foster tolerance versus immunogenic mature DCs, inhibiting maturation—for instance via IL-38-emerges as a viable therapeutic avenue (6).

#### 3.2.5 Natural killer cells

Natural Killer (NK) cells, as cytotoxic lymphocytes of the innate immune system, play a complex role in AS. They exhibit dual functions that are highly context-dependent. On one hand, NK cells promote plaque development by secreting cytotoxic substances such as granzyme B and perforin that directly damage vascular cells (64). They also interact with DCs to enhance mutual activation and promote inflammation (53). On the other hand, NK cells possess immunoregulatory functions; surface receptors such as CD160 can mediate cytokine production that modulates disease progression (39). Upon activation, NK cells release pro-atherogenic Th1 cytokines, including IFN-γ and TNF-α, as well as Th2 cytokines such as IL-4 and IL-13, influencing plaque stability (16). Notably, NK cells produce chemokines like MIP-1α to recruit other immune cells, accelerating plaque instability (35, 42). Thus, the net effect of NK cells depends on the local microenvironment and their crosstalk with other immune effectors.

#### 3.2.6 Mast cells

Mast cells (MCs) are strategically located in the adventitia and plaque shoulder regions (16, 65). Research has demonstrated that MCs and their mediators significantly influence plaque progression and stability (65). Upon activation, MCs release potent proteases such as tryptase and chymase, alongside pro-inflammatory cytokines like TNF-α. Crucially, chymase activates latent MMPs and induces SMC apoptosis, directly degrading the fibrous cap and promoting plaque rupture (1, 65). MCs also produce mediators including IL-6 and IFN-γ that modulate inflammation, angiogenesis, and tissue remodeling (66, 67). As key initiators of the immune response, MCs contribute to cholesterol accumulation and plaque instability, marking them as critical effectors in the pathogenesis of AS.

### 3.3 Adaptive immune cells

B lymphocytes and T lymphocytes serve as the primary participants in adaptive immunity, a system that also involves NK T cells, effector T and B cells, and APCs.

#### 3.3.1 T cells and their subsets

Adaptive immunity in AS is orchestrated by distinct T cell subsets (15). Th1 cells drive inflammation and plaque instability by secreting cytokines including IFN-γ, TNF-α, and IL-2, which activate macrophages and inhibit SMC collagen synthesis (5, 8, 15, 28). Conversely, Th2 cells exhibit complex effects: while IL-4 plays a dual role, IL-5 and IL-13 are predominantly protective (15). Th17 cells amplify inflammation by recruiting neutrophils via IL-17 and IL-22, facilitating early-stage matrix remodeling (15). FoxP3+ Tregs maintain plaque stability by secreting IL-10 and TGF-β to suppress effector T cells and promote M2 macrophage polarization; their depletion accelerates disease progression (8, 36, 68). In contrast, cytotoxic CD8+ T cells induce macrophage apoptosis via perforin/granzyme and FasL, expanding the necrotic core and instability (33, 67). Accordingly, CD8+ T cell depletion ameliorates AS lesions (33).

T cell activation is governed by co-stimulatory axes. While CD28 promotes activation, CTLA-4 and PD-1 serve as negative regulators and potential therapeutic targets (68, 69). The CD40L–CD40 axis exhibits cell-specific roles, with T cell-derived CD40L driving atherogenesis (69). Furthermore, lipid metabolism modulates T cell function: ox-LDL acts as an autoantigen to drive immune responses (2), while ABCA1/ABCG1-mediated cholesterol efflux regulates TCR signaling (70). Thus, restoring the balance between pro-inflammatory (Th1, Th17, and CD8+) and protective (Treg and Th2) subsets offers a viable immunotherapeutic strategy.

#### 3.3.2 B cells

B cells are central to humoral immunity, functioning as both antibody producers and APCs (66, 67). Upon activation, they differentiate into plasma cells or internalize antigens via BCRs to prime Th1/Th2 responses (2, 71). In AS, B cell subsets exhibit dichotomous roles (15). Protective B1 cells produce innate IgM antibodies that bind oxidation-specific epitopes on LDL and apoptotic cells; this blockade inhibits macrophage lipid uptake and foam cell formation while facilitating IL-5-mediated clearance (8, 31). Similarly, marginal zone B cells and IL-10-secreting regulatory B cells attenuate inflammation and induce Tregs (15, 72). Conversely, follicular B cells exacerbate AS by secreting IgG and amplifying pro-inflammatory Th1 responses (15). Mechanistically, while IgM confers protection via neutralization and complement-mediated phagocytosis, auto-reactive IgE binds FcϵRI on myeloid cells and VSMCs to trigger inflammation (67). Additionally, B cells modulate T cell responses via lipid antigen presentation (16). Thus, the balance between these subsets dictates the immunological outcome of AS.

#### 3.3.3 Antigen-presenting cells

Antigen-presenting cells (APCs), comprising macrophages, DCs, and B cells, bridge innate and adaptive immunity to drive AS (7). B cells present self-antigens including heat shock protein 60 to naive T cells via MHC class II complexes, thereby triggering CD4+ differentiation and CD8+ cytotoxicity (7, 28). This process relies on the immunological synapse, a dynamic interface regulated by co-stimulatory signals. Specifically, the engagement of CD40 on APCs by CD40L—expressed on T cells, platelets, and ECs—amplifies inflammation by promoting DC maturation, antibody production, and leukocyte adhesion (8). Crucially, MyD88-mediated DC maturation enhances antigen presentation (31), directing naive T cell commitment into distinct lineages, including FoxP3-expressing Tregs, T-bet-expressing Th1 cells, GATA3-expressing Th2 cells, RORγT-expressing Th17 cells, and Bcl-6-expressing follicular helper T cells. Ultimately, this APC-orchestrated polarization dictates the balance between plaque stability and disease progression (70).

### 3.4 Endothelial dysfunction

**Figure 2:**
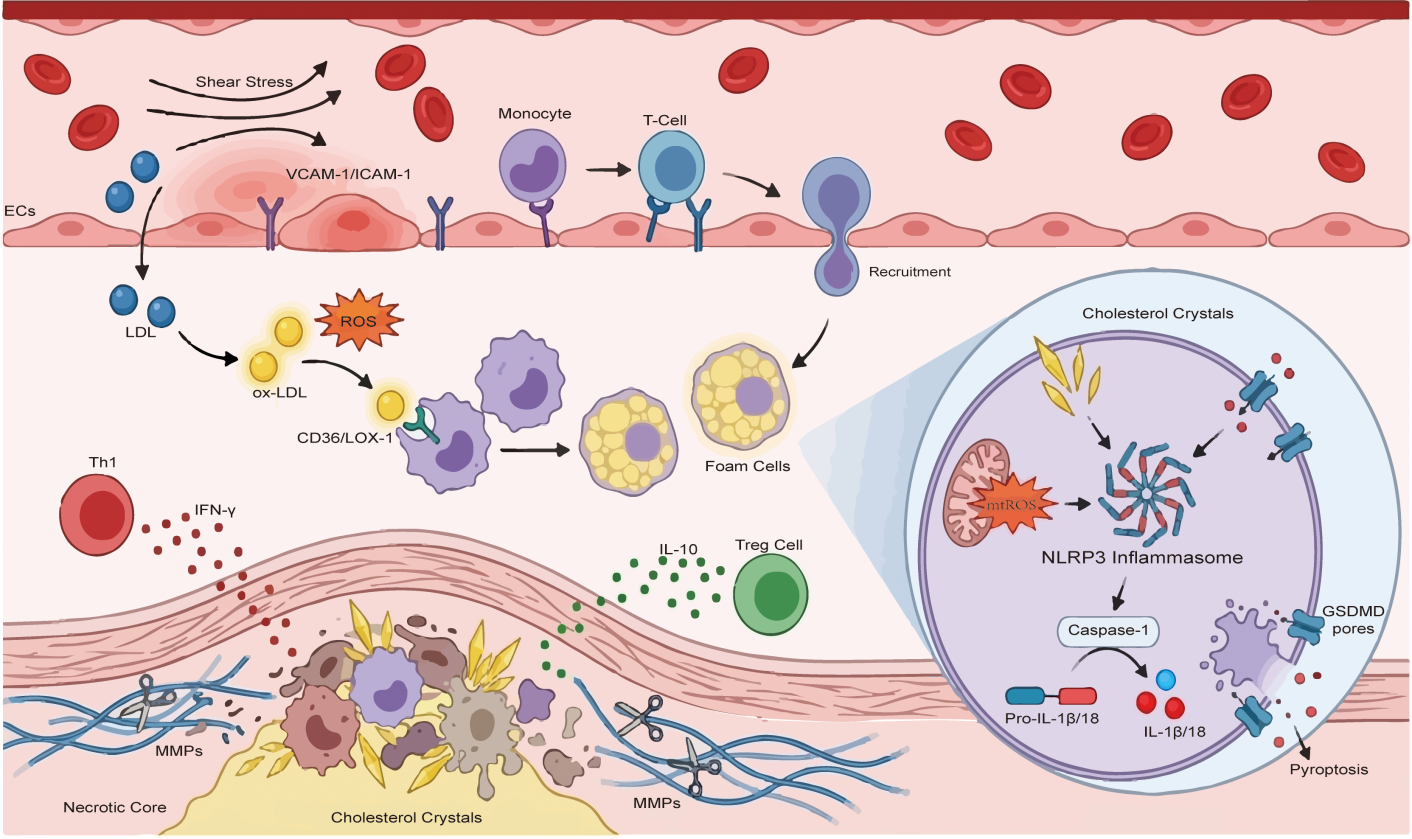
Mechanistic integration of endothelial dysfunction, oxidative stress, and NLRP3 inflammasome activation in atherosclerosis. The schematic illustrates the pathogenic cascade across the arterial wall structure. Initiation via endothelial dysfunction (Top layer). Hemodynamic shear stress triggers endothelial activation, upregulating adhesion molecules VCAM-1/ICAM-1 to facilitate leukocyte recruitment. In the subendothelial space, reactive oxygen species (ROS) modify LDL into oxidized LDL (ox-LDL). Inflammation and plaque instability (Middle layer). Macrophages actively internalize ox-LDL, mediated primarily by the scavenger receptor CD36, driving their transformation into lipid-laden foam cells. The local inflammatory milieu is sustained by the imbalance between pro-inflammatory Th1 cells and atheroprotective Treg cells. At the base, matrix metalloproteinases (MMPs) degrade the fibrous cap atop a necrotic core. Intracellular NLRP3 inflammasome activation (Inset). Within macrophages, internalized cholesterol crystals and mitochondrial ROS (mtROS) trigger the assembly of the NLRP3-ASC-Caspase-1 complex. Activated Caspase-1 cleaves pro-IL-1 and pro-IL-18 into bioactive cytokines and processes Gasdermin D (GSDMD) to form membrane pores, inducing pyroptosis and releasing inflammatory contents. VCAM-1, Vascular cell adhesion molecule 1; ICAM-1, Intercellular adhesion molecule 1; ROS, Reactive oxygen species; ox-LDL, Oxidized Low-density lipoprotein; Tregs, Regulatory T cells; MMPs, Matrix metalloproteinases; mtROS, Mitochondrial reactive oxygen species; NLRP3, NLR family pyrin domain containing 3; ASC, Apoptosis-associated speck-like protein containing a CARD.

The pathogenesis of AS is initiated by endothelial dysfunction, a critical perturbation triggered by hemodynamic disturbances including turbulent flow and shear stress, hypertension, dyslipidemia, and oxidative stress (**Figure 2**) (73). Vascular endothelial injury facilitates the subendothelial retention and oxidation of LDL (32, 74). The resultant ox-LDL activates ECs to upregulate adhesion molecules and chemokines, thereby recruiting circulating monocytes into the intima (24, 67). These infiltrated immune cells subsequently drive foam cell formation and necrotic core expansion, exacerbating the local inflammatory milieu (32, 62, 64, 71, 75).

As the disease progresses, lipid-laden foam cells and cellular debris coalesce to form a macrophage-rich necrotic core, promoting plaque expansion (6, 62). Given that ECs act as the primary interface maintaining vascular homeostasis (76), therapeutic strategies must prioritize endothelial protection. For instance, the bone marrow-derived growth factor MYDGF can remotely suppress vascular inflammation and preserve the endothelial barrier (77). Mechanistically, disturbed shear stress compromises barrier function by inducing Endothelial-to-Mesenchymal Transition via the Alk5-Shc pathway (78).

### 3.5 Oxidative stress

Oxidative stress drives the initiation and progression of AS, primarily through the subendothelial accumulation and oxidative modification of LDL (36, 64). In regions of disturbed hemodynamics, oscillatory shear stress induces a pro-inflammatory endothelial phenotype, promoting ROS-mediated generation of ox-LDL (79, 80). Acting as a potent DAMP, ox-LDL binds to the LOX-1 receptor on ECs. This interaction downregulates endothelial nitric oxide synthase (eNOS)—reducing nitric oxide bioavailability—while concurrently upregulating adhesion molecules (VCAM-1, ICAM-1) (28, 34, 79)and chemokines including CCL2, CXCL1, and CXCL8 to recruit monocytes (2, 6). Furthermore, enzymatic systems such as myeloperoxidase (MPO) generate reactive oxidants that modify ox-LDL into highly pro-inflammatory species capable of inducing IL-8 and TNF-α (81).

Following intimal entry, macrophages internalize these oxidized lipids to transform into foam cells (53, 73). This process establishes a positive feedback loop: foam cells, particularly under metabolic stress, upregulate NOX2 and MPO to generate excessive ROS and cytokines including IL-1β, TNF-α, and IL-6, which further propagate lipid peroxidation and inflammation (20, 34, 82, 83). Beyond innate immunity, ox-LDL serves as an autoantigen presented by DCs to activate CD8+ T cells (33), while also modulating CD4+ responses by enhancing Treg differentiation (68) and suppressing Th17 cells (84).

Critically, oxidative stress and resultant ROS production destabilize plaques by serving as key triggers for NLRP3 inflammasome activation and subsequent pyroptosis (25, 27, 28, 85, 86).Severe oxidative stress further disrupts homeostasis by inducing ferroptosis, an iron-dependent cell death that thins the fibrous cap and expands the necrotic core (27, 55). Conversely, natural IgM antibodies targeting ox-LDL neutralize these antigens to inhibit foam cell formation, providing an intrinsic anti-atherosclerotic defense (67).

### 3.6 The role of inflammation in AS

AS is fundamentally a chronic, immune-mediated inflammatory disease driven by both innate and adaptive immunity (14, 87). Inflammation permeates all stages of AS, from initial endothelial dysfunction (88) to plaque formation, instability, and rupture (15, 55).

#### 3.6.1 Initiation of inflammation and immune cell recruitment

The initiation of AS relies on endothelial activation triggered by DAMPs, such as ox-LDL (76, 89). Activated ECs express adhesion molecules VCAM-1 and ICAM-1 (28), which recruit circulating monocytes and T lymphocytes into the vascular wall (73, 79). Macrophages and T cells serve as the core effector cells of plaque inflammation: macrophages release pro-inflammatory cytokines, such as TNF-α, IL-1β, and IL-6 (16), to induce necrosis and amplify inflammation (90). Concurrently, Th1 cells secrete IFN-γ (91) to further activate macrophages and accelerate disease progression (31, 92). Furthermore, neutrophils, DCs, and MCs exacerbate this cascade by releasing proteases, histamine, and matrix metalloproteinases (MMPs) (73).

#### 3.6.2 Dual roles of key cytokines

AS progression is dictated by the balance between pro- and anti-inflammatory cytokine networks. Pro-inflammatory cytokines IL-1β, IL-6, and TNF-α, are the primary atherogenic drivers (87). They enhance leukocyte recruitment by activating the p38 MAPK/NF-κB (8) or JAK/STAT pathways (20). Specifically, IL-1β promotes endothelial apoptosis, foam cell formation, and plaque instability (93, 94), while IL-6 drives VSMC proliferation via the gp130 receptor (94). Additionally, IFN-γ exerts a destabilizing effect by inhibiting collagen synthesis and enhancing oxidative stress (68, 91), acting synergistically with IL-18 and IL-12 to promote matrix degradation (52). Clinically validating the inflammatory hypothesis of AS, the landmark CANTOS trial demonstrated that selectively neutralizing IL-1β with canakinumab significantly reduces recurrent cardiovascular events independent of lipid lowering (87).

Conversely, anti-inflammatory cytokines exert protective effects. IL-10, secreted by macrophages and regulatory T cells, inhibits pro-inflammatory signaling via the STAT3 pathway and promotes cholesterol efflux (30, 56). Similarly, IL-38 antagonizes the IL-1 receptor family to suppress angiogenesis, apoptosis, and NLRP3 inflammasome activation, thereby maintaining plaque stability (6).

#### 3.6.3 Regulation of inflammatory signalling pathways

The NF-κB pathway serves as a central inflammatory hub. Its activation by stimuli like ox-LDL via TLR4 drives the expression of chemokines and pro-inflammatory cytokines, promoting foam cell differentiation (28, 76). Other cascades concurrently modulate inflammation: the JAK/STAT pathway enhances cytokine release (20, 92), while PI3K/Akt signaling regulates macrophage polarization and autophagy (20). Furthermore, epigenetics play a crucial regulatory role; for example, ox-LDL upregulates DNMT1, which suppresses the protective endothelial gene KLF2 via DNA methylation (21).

#### 3.6.4 Inflammation and plaque instability

Advanced AS features plaque instability, a primary precursor to acute cardiovascular events. Mediators like IFN-γ, TNF-α, and IL-1β induce VSMC apoptosis, inhibit collagen synthesis, and activate MMP-2 and MMP-9 to degrade the fibrous cap (15, 92). Concurrently, NLRP3 inflammasome-driven pyroptosis exacerbates the expansion of the necrotic core by releasing inflammatory contents (25, 76, 85, 95). Extracellular matrix degradation combined with secondary necrosis further promotes thrombosis (55, 88). Notably, IL-10 deficiency impairs the resolution of inflammation, thereby perpetuating chronic inflammatory states and provoking persistent tissue damage (30). Clinical translation underscores that therapeutic precision is paramount. While targeted IL-1β inhibition succeeded in the CANTOS trial, broad-spectrum immunosuppression via low-dose methotrexate failed in the CIRT trial (94). Ultimately, future therapeutic strategies must move beyond generalized immunosuppression to precisely target specific inflammatory components, notably the NLRP3 inflammasome and its downstream effectors.

### 3.7 Inflammasomes and AS

Inflammasomes are multiprotein innate immune complexes that recognize PAMPs and DAMPs to drive inflammation (96, 97). Among these, the NLRP3 inflammasome emerges as a pivotal driver of AS pathogenesis (39). Structurally, this tripartite complex comprises the sensor NLRP3, the adaptor ASC, and the effector pro-caspase-1. The NLRP3 sensor itself features an amino-terminal pyrin domain, a central NACHT domain, and a carboxy-terminal ligand-sensing leucine-rich repeat domain (22, 55, 98, 99).

#### 3.7.1 Two-step activation mechanism

NLRP3 activation requires a strictly regulated two-step process. First, a “priming signal” from TLR ligands or TNF-α triggers NF-κB-mediated upregulation of NLRP3, pro-IL-1β, and pro-IL-18 (5, 92). Second, an activation signal driven by potassium efflux, mitochondrial dysfunction, or lysosomal destabilization directly promotes the oligomeric assembly of the NLRP3-ASC-caspase-1 tripartite complex (97, 99). Subsequently, caspase-1 cleaves pro-IL-1β and pro-IL-18 into mature cytokines and processes gasdermin D to induce pyroptosis, profoundly amplifying the inflammatory cascade (22, 55, 100).

#### 3.7.2 Pathological consequences

In AS, NLRP3 activation exhibits distinct, cell type-specific roles rather than a generalized inflammatory response (101).In macrophages, NLRP3 activation is driven by subendothelial ox-LDL and cholesterol crystals acting as DAMPs (39, 98). Phagocytosed via CD36, these agents activate the NLRP3 inflammasome through a CD36-TLR4-TLR6 signaling complex, triggering lysosomal destabilization and mitochondrial ROS generation (28, 97, 102). Highly expressed in plaque macrophages (103), Highly expressed in these plaque macrophages (102), activated NLRP3 promotes necrotic core expansion and plaque rupture primarily via GSDMD-mediated pyroptosis (43, 85). Conversely, in ECs, NLRP3 is primarily activated by disturbed shear stress and metabolic factors. Rather than inducing immediate pyroptosis, endothelial NLRP3 activation predominantly leads to endothelial dysfunction and the upregulation of adhesion molecules VCAM-1 and ICAM-1, thereby facilitating early leukocyte recruitment (104, 105).

Furthermore, it is crucial to recognize that IL-1 signaling in AS is not exclusively inflammasome-dependent. While the NLRP3/caspase-1 axis is a major source of mature IL-1β, pro-IL-1β and pro-IL-1α can also be cleaved into their bioactive forms by neutrophil-derived serine proteases or mast cell chymase, bypassing the canonical inflammasome assembly (101). This inflammasome-independent IL-1 processing sustains the local inflammatory milieu and matrix degradation even when classical NLRP3 signaling is pharmacologically inhibited. Thus, targeting the NLRP3 inflammasome using agents like MCC950 stabilizes plaques (45, 106, 107), but future precision therapies must account for both cell-specific roles and alternative IL-1 cleavage pathways (106, 108).

## 4 Myocarditis

### 4.1 Introduction

Myocarditis is an inflammatory disease of the myocardium with a heterogeneous etiology. The most prevalent pathogen is Coxsackievirus B3 (CVB3), followed by adenovirus, parvovirus B19, and human herpesviruses (24). Enteroviruses, particularly CVB3, account for 15%–30% of acute myocarditis cases (109). Its pathogenesis primarily involves direct viral injury and the subsequent host immune response. The disease course is typically conceptualized as a triphasic continuum: the viral replication phase, where the virus invades and activates innate immunity; the autoimmune phase, where adaptive immunity is triggered; and the chronic phase, where persistent inflammation leads to myocardial remodeling, fibrosis, and potential progression to dilated cardiomyopathy (DCM) and heart failure (35, 71).

The immune response in myocarditis exhibits a dichotomous nature: while a moderate response facilitates pathogen clearance, an excessive or dysregulated response exacerbates cardiac injury, potentially triggering fulminant myocarditis or chronic inflammatory cardiomyopathy (4). The global incidence of viral myocarditis is approximately 10–22 per 100, 000 individuals, with CVB3 infection accounting for over 50% of confirmed cases (110). Notably, autoimmune responses may persist even after viral clearance, driving chronic disease. Based on the infiltrating cell type, myocarditis is classified into subtypes including lymphocytic, giant cell, and eosinophilic myocarditis (109, 111). Lymphocytic myocarditis is the most common, arising from viral infections, autoimmune diseases, or Immune Checkpoint Inhibitors (ICIs) (112). ICI-induced myocarditis represents a distinct, high-mortality entity characterized by the expansion of a specific CD8+ effector memory T cell subset, Temra, that re-expresses CD45RA and exhibits a cytotoxic phenotype (113, 114). Furthermore, in ICI myocarditis, a pathogenic feed-forward loop exists where CD8+ T cells producing IFN-γ induce CCR2+ macrophages to express CXCL9 and CXCL10, thereby amplifying inflammation (115). Despite its unique triphasic progression, the core immunopathogenesis of myocarditis converges on common cardiovascular mechanisms involving innate and adaptive immunity, orchestrated by complex cytokine networks, the activation of inflammasomes, and targets for emerging immunotherapies.

### 4.2 Viral phase

#### 4.2.1 Viral invasion, replication, and host innate immune response

**Figure 3:**
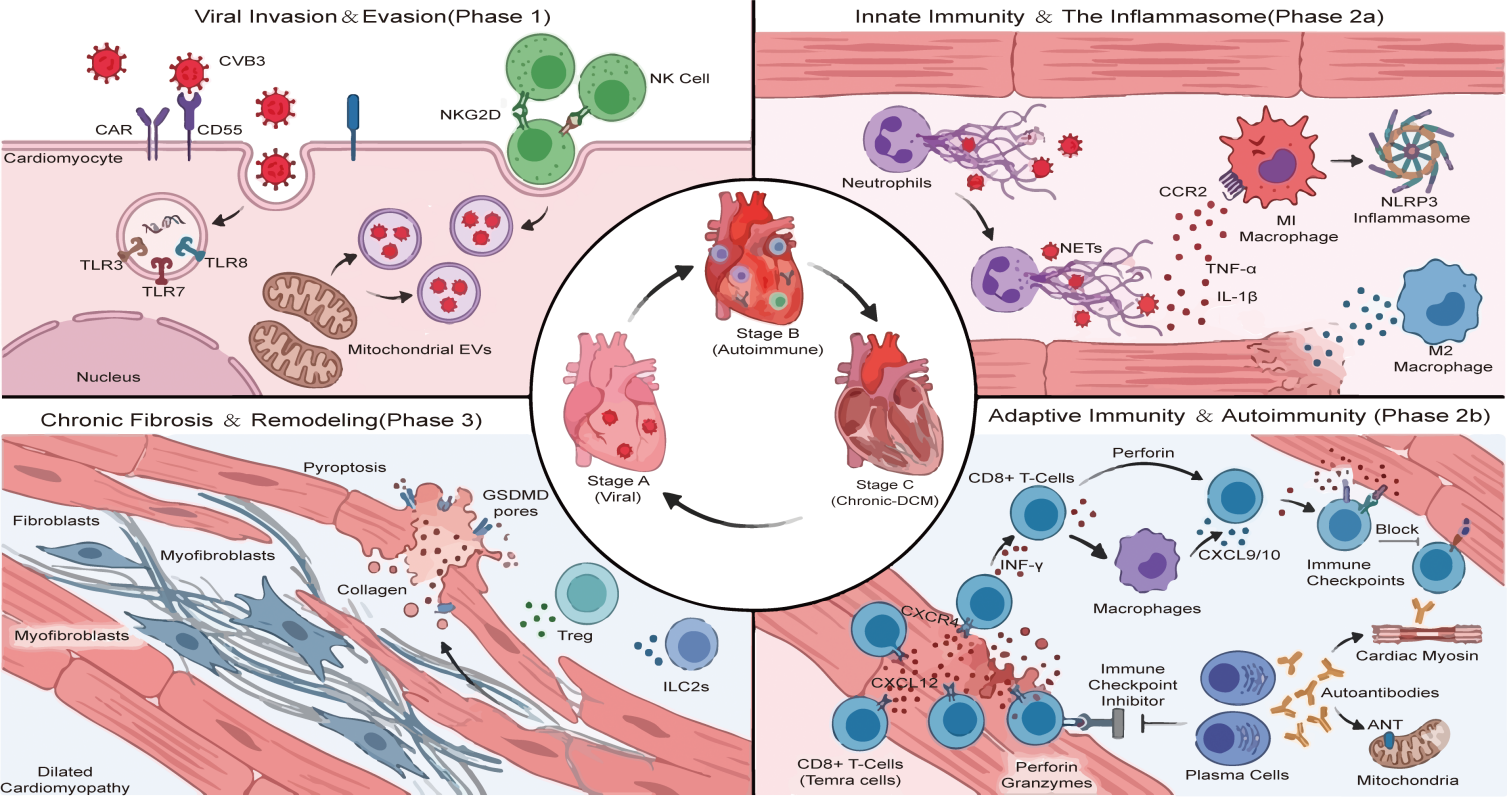
The immunopathogenesis of myocarditis progression: from viral entry to chronic remodeling. The diagram illustrates the temporal evolution of myocarditis across three distinct phases surrounding a central disease progression cycle. Phase I: Viral invasion and evasion. Coxsackievirus B3 (CVB3) enters cardiomyocytes via CAR and CD55 receptors. Intracellular viral RNA is detected by Toll-like receptors TLR3, TLR7/8. Mitochondria release virus-containing extracellular vesicles (EVs) to facilitate evasion, while Natural Killer (NK) cells target infected cells via NKG2D signaling. Phase IIa: Innate immunity and inflammasome activation. Neutrophils release neutrophil extracellular traps (NETs). Pro-inflammatory M1 macrophages, recruited via CCR2, secrete TNF-α and IL-1β while activating the NLRP3 inflammasome. M2 macrophages exhibit an anti-inflammatory phenotype. Phase IIb: Adaptive immunity and autoimmunity. CD8 T cells release perforin and granzymes to induce cytotoxicity. A feed-forward loop involving IFN-γ and CXCL9/10 sustains macrophage-T cell interaction. Plasma cells produce autoantibodies targeting cardiac myosin and the adenine nucleotide translocator (ANT). Immune checkpoint blockade exacerbates T cell activation. Phase III: Chronic fibrosis and remodeling. Persistent inflammation drives the transition of fibroblasts and myofibroblasts, leading to excessive collagen deposition. Gasdermin D (GSDMD)-mediated pyroptosis contributes to cell death. Despite regulation by Tregs and Type 2 innate lymphoid cells (ILC2s), maladaptive remodeling ultimately results in dilated cardiomyopathy (DCM). CVB3, Coxsackievirus B3; CAR, Coxsackievirus and adenovirus receptor; TLR, Toll-like receptor; EVs, Extracellular vesicles; NKG2D, Natural killer group 2D; NETs, Neutrophil extracellular traps; CCR2, C-C chemokine receptor type 2; NLRP3, NLR family pyrin domain containing 3; CXCL, C-X-C motif chemokine ligand; ANT, Adenine nucleotide translocator; GSDMD, Gasdermin D; ILC2s, Group 2 innate lymphoid cells; DCM, Dilated cardiomyopathy.

Viral invasion initiates pathogenesis. CVB3 enters cardiomyocytes and ECs by binding to the Coxsackievirus-Adenovirus Receptor and the co-receptor CD55 (24, 109, 116), subsequently utilizing viral proteases 2A and 3C for replication—a process intricately regulated by host non-coding RNAs (109, 117). Following invasion (**Figure 3**, Phase 1), PRRs activate innate immunity. TLR7 and TLR8 recognize single-stranded viral RNA to upregulate cytokines including TNF-α, IL-6, and IFN-γ, alongside chemokines such as CCL2 and CCL20, recruiting NK, γδ T, and CD8+ T cells for early defense (71, 109). Crucially, TLR3 senses double-stranded RNA intermediates; its signaling via the TRIF-TRAF6-NF-κB axis is essential for controlling replication (118). Conversely, MyD88-mediated TLR4 activation promotes IL-1β/IL-18 release to exacerbate injury (119).

Additionally, CVB3 triggers the NLRP3 inflammasome, driving caspase-1-dependent pyroptosis and cytokine maturation to intensify inflammation and fibrosis (120). The pathophysiological impact of NLRP3 activation is highly cell-type specific (121). In cardiomyocytes, viral replication triggers NLRP3-driven pyroptosis, causing irreversible myocardial loss and contractile dysfunction. Conversely, in macrophages and endothelial cells, NLRP3 primarily amplifies the paracrine cytokine storm. Moreover, early inflammation is exacerbated by inflammasome-independent mechanisms: proteases from necrotic cardiomyocytes and neutrophils directly cleave IL-1 family cytokines, bypassing the canonical NLRP3-ASC-Caspase-1 axis to intensify acute inflammation and fibrotic remodeling (121).

#### 4.2.2 Viral immune evasion and host factor regulation

Viruses have evolved strategies to exploit host machinery and evade clearance. Cardiotropic viruses, including CVB3, influenza, and SARS-CoV-2, target mitochondria for replication and release viral particles via mitochondrial extracellular vesicles to escape recognition (122). Paradoxically, CVB3-induced mitochondrial fission generates vesicles that activate the TLR4/NLRP3 pathway, fueling inflammation (122). Other pathogens, such as HHV-6B, suppress immunity via distinct receptors including CD134 and Nectin 2 (109). On the host side, CVB3 upregulates macrophage CAPN4 expression to promote replication (123), while infected cardiomyocytes secrete CXCL12 to recruit immune cells (124). Thus, CVB3-induced injury results from synergistic viral cytotoxicity, inflammatory cascades, and consequent tissue destruction (116).

### 4.3 Autoimmune phase

Following viral entry, if the pathogen is not completely cleared by the innate response, the disease progresses to the autoimmune phase. At this stage, adaptive immunity becomes the dominant driver, with T cell and B cell-mediated responses serving as key factors in myocardial injury (**Figure 3** Phase 2a).

#### 4.3.1 Monocytes and macrophages

Macrophages dictate the early inflammatory trajectory of myocarditis. Specifically, CCR2+ macrophages assume a pro-inflammatory M1 phenotype releasing IL-1β and IL-6, whereas CCR2- macrophages exhibit a reparative M2 phenotype essential for regeneration (51, 125). This polarization is mechanistically regulated; for instance, CVB3-induced CAPN4 drives M1 polarization via STAT1/STAT3 signaling and NLRP3 activation (123). Conversely, inhibiting miR-155 or miR-30a-5p favors M2 polarization, which confers cardioprotection via exosomal transfer of lncRNA AK083884 (116).

Recruitment is driven by infected cardiomyocytes transmitting ER stress and secreting chemokines including MCP-1 and MIP-1α to attract monocytes via CCR2/CCR5 (35, 126). Notably, KLF10 downregulation in cardiomyocytes unleashes MCP-1 expression, exacerbating Ly6C-high monocyte infiltration (119, 127). In ICI-associated myocarditis, monocytes overexpressing S100A8/A9 predominate (112). Furthermore, cardiac-infiltrating CCR2+ macrophages establish a pathogenic feed-forward loop with T cells: stimulated by T cell-derived IFN-γ, they secrete CXCL9 and CXCL10 to amplify Th1 recruitment (115).

#### 4.3.2 Neutrophils

During the acute phase, neutrophils infiltrate the myocardium and exacerbate inflammation upon TLR8-mediated recognition of CVB3 (118, 128). They release proteases, ROS, and chemokines including CXCL1 and CXCL2 (128). Crucially, NETs activate macrophages via the inflammasome to trigger a cytokine storm of TNF-α, IL-1β, and IL-18, creating a self-perpetuating cycle of injury (128).

#### 4.3.3 NK cells and other innate immune cells

NK cells prevent progression to DCM by mediating viral clearance via NKG2D and CXCL10, a function modulated by FoxO3 and miR-155 (35). In ICI myocarditis, a specific IL-32+HLA-DR+ NK subset expands, suggesting antigen-presenting capabilities (112). Additionally, CCL17 expressed by CCR2+ macrophages and DCs enhances antiviral immunity by inhibiting Treg recruitment (129), while the HMGB1-RAGE axis upregulates TLR2/4 signaling (129). Interestingly, non-immune cells also contribute; membrane-bound TNF-α on cardiac microvascular ECs facilitates leukocyte recruitment (130).

#### 4.3.4 T-cell response

Persistent pathogen exposure triggers adaptive immunity (**Figure 3**, Phase 2b), where myocardial injury and self-antigen exposure drive CD4+ T cell-mediated autoimmunity (35). Differentiation of naive CD4+ T cells is governed by cytokines and TLRs; for example, TLR2/1 signaling induces Th1/Th17 subsets, whereas TLR2/6 promotes IL-10-producing Tregs (118).

Temporally, a dominant Th1 response aids initial viral clearance but exacerbates acute inflammation, while a subsequent shift towards Th2 responses drives chronic remodeling and DCM (118). The Th17/Treg balance is critical: CVB3 disrupts this by suppressing Nup98 and upregulating miR-155 to favor fibrotic Th17 responses over protective Tregs (118).

Cytotoxic CD8+ T cells are markedly activated, upregulating perforin and IFN-γ via CXCL12/CXCR4 signaling to target both viral and self-epitopes (124). This cytotoxicity is hyperbolic in ICI-induced myocarditis, where checkpoint blockade expands CD8+ clones recognizing the α-myosin autoantigen, specifically targeting epitopes including MYH6 191–198 (113). Triggered by this initial activation, these cells drive fulminant necrosis through a pathogenic feed-forward loop: T cell-derived IFN-γ stimulates macrophages to secrete CXCL9 and CXCL10, which in turn incessantly recruit more autoreactive T cells into the myocardium (109, 113, 115). Tregs play a complex role, potentially delaying early viral clearance while being essential for limiting chronic fibrosis (126, 129).

#### 4.3.5 B cells and autoantibodies

B cells drive the adaptive phase via autoantibody production, mediating cytotoxicity and immune complex deposition (4). Autoantibodies targeting mitochondrial antigens including the adenine nucleotide translocator and cardiac proteins such as myosin and troponin are frequently detected, implicating epitope spreading or molecular mimicry (110, 122, 126). In ICI myocarditis, single-cell BCR sequencing confirms active, antigen-driven clonal expansion (112).

#### 4.3.6 Immunometabolism and other cytokine regulation

Immunometabolic reprogramming underpins pathogenesis. Phosphoglycerate kinase 1 (PGK1) promotes glycolysis to enhance Th17 differentiation and autoimmunity (131). Furthermore, T cell-induced mitochondrial dysfunction releases mtDNA, which acts as a DAMP to persistently activate TLR9 and pathogenic Th1 responses (132, 133). This milieu is fine-tuned by cytokines: IL-1β serves as a therapeutic target, while TNF-α negatively regulates autoreactive T cells via activation-induced cell death (120, 130).

### 4.4 Chronic phase and cardiac remodelling

#### 4.4.1 Mechanisms of chronic inflammation and myocardial fibrosis

Persistent inflammation drives the progression to DCM and heart failure, histologically characterized by fibrosis and immune infiltration (**Figure 3**, Phase 3) (4, 111). Mechanistically, this chronic phase is sustained by endogenous ligands; specifically, cardiac myosin peptides released from damaged tissue stimulate TLR2/8 to drive Th17 responses (118). Concurrently, sustained NLRP3 activation and inflammasome-independent IL-1β processing synergistically drive NF-κB-mediated fibroblast activation (4, 121). Specific molecular regulators modulate this remodeling process: gasdermin D-mediated pyroptosis facilitates the transition of fibroblasts to myofibroblasts (120), whereas macrophage-derived CAPN4 inhibits collagen synthesis via paracrine signaling (123). Conversely, the transcription factor KLF10 exerts a protective effect, limiting fibrosis by suppressing MCP-1 expression (127).

#### 4.4.2 Immunoregulation and therapeutic strategies

The triphasic nature of myocarditis demands phase-specific interventions that balance viral clearance against chronic damage (14, 71). During this phase, group 2 innate lymphoid cells in pericardial adipose tissue promote repair via Treg recruitment and M2 macrophage polarization (134), whereas ICI-associated T cells may undergo exhaustion (114). Specifically, ICIs disrupt the normal PD-1/PD-L1 barrier within the heart, leading to clonal expansion of CD8+ T cells recognizing cardiac-tumor shared antigens such as myosin. This ultimately induces fulminant myocardial necrosis (135, 136). Therapeutically, managing viral myocarditis requires combining antiviral and immunomodulatory agents (109). Dictated by the triphasic pathogenesis, broad immunosuppression is contraindicated during early viral replication but specifically warranted for the subsequent autoimmune and chronic phases.For ICI-induced myocarditis, disrupting inflammatory feed-forward loops is critical; strategies include targeting the IFN-γ pathway with JAK inhibitors such as ruxolitinib or restoring checkpoints using abatacept (115). Broader anti-inflammatory approaches show efficacy: interleukin-1 receptor blockade with anakinra mitigates structural damage (4), while colchicine inhibits the NLRP3 inflammasome (119). Additionally, mesenchymal stem cells exert paracrine immunomodulation to block apoptosis and viral replication (116). Future precision strategies, including immunoadsorption and gene editing, aim to restore cardiac immune homeostasis.

## 5 Vasculitis

### 5.1 Introduction

Vasculitis comprises heterogeneous disorders characterized by vascular inflammation and necrosis causing ischemia or aneurysms (137). Clinically stratified by vessel caliber, the spectrum includes large-vessel vasculitis such as giant cell arteritis (GCA), medium-vessel variants including Kawasaki disease (KD), and small-vessel entities exemplified by ANCA-associated vasculitis (AAV) (23, 138). These conditions share a pathogenic breakdown of vascular immune privilege (23, 138, 139). While classically viewed as autoantibody-driven, emerging evidence highlights the pivotal role of innate immune dysregulation in initiating this breach (140).

**Figure 4:**
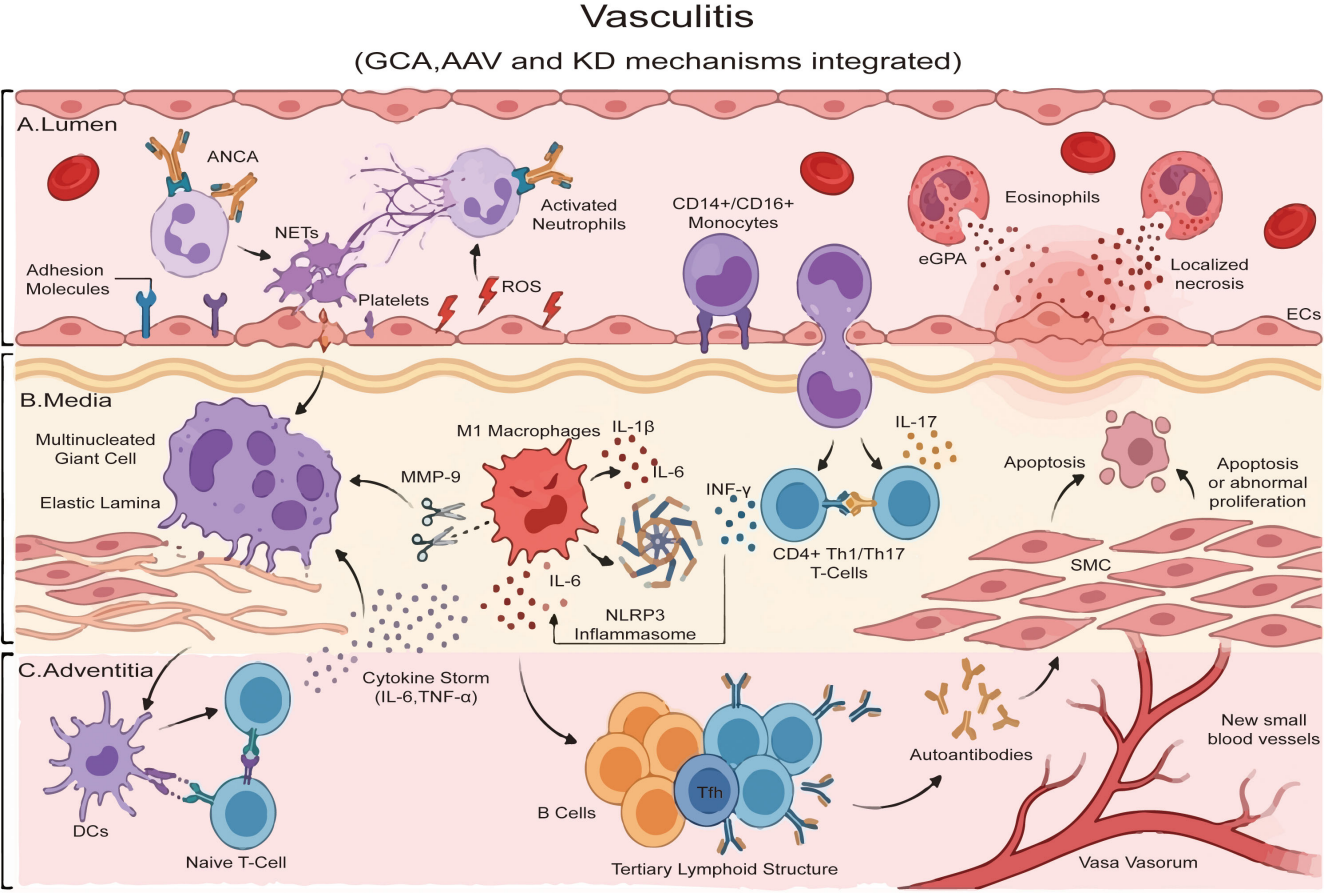
Integrated immunopathogenesis of systemic vasculitides across the arterial wall. While these distinct diseases rarely co-occur clinically within a single vessel, they are conceptually integrated here to highlight their layer-specific immune mechanisms.The schematic illustrates the distinct yet overlapping immune mechanisms of Giant Cell Arteritis (GCA), ANCA-associated vasculitis (AAV), and Eosinophilic Granulomatosis with Polyangiitis (eGPA) within a stratified vessel cross-section. **(A)** Lumen. This layer highlights mechanisms prominent in AAV and eGPA. Anti-neutrophil cytoplasmic antibodies (ANCA) activate neutrophils, triggering the release of reactive oxygen species (ROS) and neutrophil extracellular traps (NETs). Eosinophils degranulate to release cytotoxic proteins, while CD14+/CD16+ monocytes adhere to activated endothelial cells expressing adhesion molecules. **(B)** Media. This layer depicts hallmark features of GCA. Infiltrating monocytes differentiate into M1 macrophages and fuse to form multinucleated giant cells, which degrade the elastic lamina via matrix metalloproteinases (MMPs). The NLRP3 inflammasome drives IL-1β and IL-6 production. CD4+ Th1 and Th17 cells sustain inflammation via IFN-γ and IL-17 signaling, leading to smooth muscle cell (SMC) apoptosis or abnormal proliferation. **(C)** Adventitia. Dendritic cells (DCs) present antigens to naive T cells, initiating the adaptive response. Tertiary lymphoid structures (TLS) containing B cells and T follicular z cells facilitate local autoantibody production. Neovascularization supports continuous immune cell infiltration. GCA, Giant cell arteritis; eGPA, Eosinophilic granulomatosis with polyangiitis; AAV, ANCA-associated vasculitis; ANCA, Anti-neutrophil cytoplasmic antibody; MMPs, Matrix metalloproteinases; SMCs, Smooth muscle cells; DCs, Dendritic cells; TLS, Tertiary lymphoid structures.

In GCA, pathogenesis follows a multi-step trajectory involving innate-adaptive interplay (**Figure 4**). An asymptomatic phase of innate dysregulation precedes tolerance breach and the translocation of peripheral effectors to the adventitia, triggering a granulomatous attack that overwhelms vascular protection (141, 142). Specifically, adventitial dendritic cell activation orchestrates a chemokine-mediated influx of T cells and monocytes to compromise vascular integrity (142, 143). Crucially, inflammation acquires autonomy via molecular checkpoints including aberrant Notch signaling, PD-1/PD-L1 defects, and self-renewing tissue-resident memory T cells (144, 145). This autonomy drives therapeutic resistance and a transcriptional signature defined by monocyte activation (144, 145). Similarly, in KD, the NLRP3 inflammasome and downstream IL-1β act as key drivers of coronary arteritis (96). The progression of vasculitis is intricately driven by the interplay between innate and adaptive immunity, marked by robust immune cell infiltration, specific cytokine storms, and inflammasome activation, which collectively inform modern targeted therapies.

### 5.2 Immunological mechanisms of vasculitis

The destruction of the vascular wall is orchestrated by a complex crosstalk between innate sensors and adaptive effectors, with specific mechanisms varying across vasculitis subtypes.

#### 5.2.1 Immune cell infiltration

In vasculitis, the vascular wall is infiltrated by CD4+ T cells, DCs, macrophages, and multinucleated giant cells (141), which secrete MMPs and cytokines including IL-6 and TNF-α to disrupt vascular architecture (23). Distinct DC subsets drive inflammation initiation: monocyte-derived DCs produce IL-12 and IL-1β to activate B cells, whereas classical DCs specialize in antigen presentation to efficiently stimulate CD4+ and CD8+ T cells (146).

#### 5.2.2 Neutrophils

Neutrophil activation and NETosis play context-dependent roles (147). In AAV, neutrophils bound by PR3-ANCA or MPO-ANCA autoantibodies undergo Fcγ receptor-mediated activation, releasing ROS and forming NETs that directly injure ECs (148, 149). These NETs further exacerbate disease by exposing MPO/PR3 autoantigens and activating DCs to breach tolerance (147). In GCA, immature neutrophils including band cells and myelocytes exhibit defective NETosis yet produce excessive ROS to compromise the endothelial barrier (150). Similarly, spontaneous NET formation drives vascular damage in KD, whereas eosinophils replace neutrophils as the primary source of toxic granule proteins in Eosinophilic granulomatosis with polyangiitis (eGPA) (151).

#### 5.2.3 Monocytes and macrophages

Monocytes and macrophages drive GCA and TAK pathogenesis. Expanded CD14+CD16+ monocytes expressing TLR2/4 and HLA-DR are primed to produce cytokines including IL-1β, IL-6, and TNF-α (152). In GCA, recruited monocytes undergo glycolytic reprogramming to establish trained immunity, which sustains inflammation through enhanced glucose uptake and IL-6 production (153). Phenotypically, macrophages evolve spatially: GM-CSF-driven CD206-MMP-9+ macrophages mediate early tissue destruction, whereas M-CSF-driven FRβ+ macrophages later promote fibrosis and neovascularization via PDGF (152). Systemically, bone marrow “emergency hematopoiesis” responds to vascular DAMPs by releasing activated myeloid cells (144). In AAV, macrophage uptake of PR3-expressing apoptotic neutrophils impairs M2-like clearance, triggering cytokine release that recruits additional leukocytes (154). As innate effectors, NK cells potentially contribute to AAV pathogenesis through cytokine production or direct cytotoxicity (155).

#### 5.2.4 NLRP3 inflammasome and other regulatory factors

The NLRP3 inflammasome links metabolic stress to vascular inflammation, activated by stimuli including cholesterol crystals, ox-LDL, and hypoxia (96). Emerging evidence indicates that long non-coding RNAs regulate this process; for instance, TNF-α upregulates endothelial MIR181A1HG to promote activation and monocyte adhesion via NLRP3 (156).

#### 5.2.5 T cells

Vascular DCs drive a rigid polarization of T cell responses (146). Large-vessel vasculitis (GCA and TAK) favors Th1 and Th17 phenotypes: IFN-γ-secreting Th1 cells drive granuloma formation, while IL-17-secreting Th17 cells promote remodeling (146, 157). Conversely, eGPA exhibits a Th2 dominance driven by IL-4, IL-5, and IL-13 (158). Distinctly, single-cell transcriptomics in GCA reveals expanded cytotoxic CD4+ T cells expressing perforin and granzyme B that contribute to vascular death (159), whereas TAK features a T follicular helper signature implicating B cell interaction (160).

Mechanistically, pro-inflammatory polarization involves JAK-STAT and mTORC1 pathways (157), alongside a loss of self-tolerance. In GCA, Notch1+ CD4+ T cells expand, while aberrant Notch4 signaling in Tregs disrupts immunosuppressive exosome release (144). Although central to tolerance, Treg numbers and function are compromised in GCA and AAV (159, 161). Under IL-6-rich conditions, Tregs may transdifferentiate into pathogenic Th17-like cells (162), while Hedgehog-driven ZFYVE21 activation further promotes NLRP3-mediated inflammation (163).

#### 5.2.6 B cells

B cells exhibit multifaceted roles beyond autoantibody production. In GCA and TAK, they organize into adventitial tertiary lymphoid structures. While peripherally quiescent, these tissue-resident B cells actively secrete cytokines including IL-6, GM-CSF, and TNF-α to modulate macrophage phenotypes within the granulomatous niche (143, 145). In contrast, B cell pathology in AAV is primarily driven by the production of ANCA autoantibodies.

### 5.3 The role of inflammation in vasculitis

Vasculitis is characterized by a systemic inflammatory response involving elevated levels of IL-1β, IL-6, TNF, and CRP, which promote leukocyte adhesion and accelerate secondary AS (23). Macrophages orchestrate this environment by secreting chemokines such as CXCL9, CXCL10, and CCL2 to recruit T cells and monocytes (152). The inflammatory milieu stimulates MMP secretion, particularly MMP-9 derived from monocytes, which digests the elastic lamina and basement membranes, facilitating immune invasion and aneurysm formation (144). In GCA, B cell-derived cytokines shape the local environment, potentially perpetuating inflammation by polarizing infiltrating and resident cells (143). Furthermore, NLRP3 inflammasome-driven IL-1β release acts as a central orchestrator, inducing endothelial adhesion molecules and synergizing with IL-18 to amplify the Th1 response (96, 156).

This leads to a pivotal question in cardiovascular immunology: despite distinct cellular compositions, do AS, myocarditis and vasculitis share a convergent molecular etiology?

## 6 Key immunological hubs and therapeutic interventions

Emerging transcriptomic evidence highlights a convergent immunological signature shared across AS, myocarditis and vasculitis (164–166). This broad pathogenic interplay of innate and adaptive immunity represents a critical driver in cardiovascular diseases (167). Recent bioinformatics analyses and multi-omics profiling have robustly identified co-hyperactivation of core immune genes across these distinct cardiovascular pathologies, specifically isolating PYCARD (168), HSPA1A, CXCR4 (169), and TSC22D3 as critical nodes (170, 171).

Rather than functioning as ubiquitous end-stage cytokines like IL-6 or TNF-α, PYCARD, CXCR4, TSC22D3, and HSPA1A are identified by transcriptomic and PPI algorithms as upstream regulatory nodes. Crucially, WGCNA demonstrates their high betweenness centrality across these cardiovascular pathologies (168–171). These molecules represent the critical intersection of innate and adaptive dysregulation, serving as molecular hubs that regulate the transition from acute inflammation to chronic remodeling.

### 6.1 Shared expression profiles and common downstream targets

The designation of CXCR4, PYCARD, TSC22D3, and HSPA1A as central immunological hubs is supported by their consistent expression profiles and convergent downstream signaling across cardiovascular pathologies. Spatially, CXCR4 is upregulated in vulnerable atherosclerotic plaques, infected myocardium, and vasculitic lesions, orchestrating systemic leukocyte trafficking (172). Similarly, PYCARD localizes to lipid-laden macrophages, infected cardiomyocytes, and necrotizing vasculature, where it drives NLRP3 inflammasome assembly, IL-1β/IL-18 maturation, and pyroptosis (170).

Conversely, immune tolerance relies on regulatory hubs like TSC22D3 and HSPA1A. TSC22D3 functions as an endogenous glucocorticoid-responsive brake, inhibiting NF-κB and MAPK pathways to govern inflammatory cell fate and promote Treg differentiation (171). Meanwhile, HSPA1A exerts dual functions: intracellular HSPA1A dampens inflammation, whereas extracellular HSPA1A acts as a systemic danger signal correlating with vascular stress and chronic inflammation (173). Together, these hubs converge onto shared effectors to form a unified immunopathological network (**Table 1**).

**Table 1:** Summary of representative therapeutic agents targeting key immune hubs.

Core target	Disease expression	Associated pathway	Therapeutic agent & Clinical status	Mechanism of action	Reference
CXCR4	Highly expressed in AS plaques, viral myocardial infiltrates, and inflamed vasculitic lesions	Leukocyte trafficking. Orchestrates chemotaxis and progenitor cell homing via the DOCK-RAC2 pathway and CXCL12 axis	Plerixafor(FDA-Approved)	Antagonizes CXCR4 to inhibit leukocyte chemotaxis towards inflamed tissues	(174)
PYCARD	Upregulated in foam cells (AS), infected myocardium, and necrotizing vasculitis	NLRP3 Inflammasome Activation. Assembles via PYD/CARD domains to recruit Caspase-1, driving IL-1β release and pyroptosis	Lycorine(Experimental)	Binds to PYCARD/ASC to disrupt inflammasome assembly and downstream pyroptosis	(175)
TSC22D3	Downregulated or overwhelmed during the chronic progression of AS, myocarditis, and vasculitis.	Immunomodulation and Cell Fate. Acts as a molecular brake to suppress the NF-κB and MAPK signaling pathways	Dexamethasone(FDA-Approved)	Exerts immunomodulatory effects by inhibiting the NF-κB signaling pathway	(176)
HSPA1A	Upregulated intracellularly for defense; extracellular release correlates with severe vascular risk in AS and vasculitis	Proteostatic Defense & Systemic Stress. Intracellularly suppresses NF-κB activation; extracellularly acts as a danger-associated molecular pattern	Tanespimycin(Investigational)	Inhibits HSP90 to trigger HSF1-mediated upregulation of *HSPA1A*, suppressing NF-κB signaling.	(177)

Agents listed represent pharmacological modulators with experimentally validated effects on the respective targets as reported in the literature.

ASC, Apoptosis-associated speck-like protein containing a CARD (encoded by *PYCARD*); CXCR4, C-X-C chemokine receptor type 4; HSP90, Heat shock protein 90; HSPA1A, Heat shock protein family A (Hsp70) member 1A; HSF1, Heat shock factor 1; NF-κB, Nuclear factor kappa B; TSC22D3, TSC22 domain family member 3 (also known as GILZ).

### 6.2 The CXCR4/CXCL12 axis

**Figure 5:**
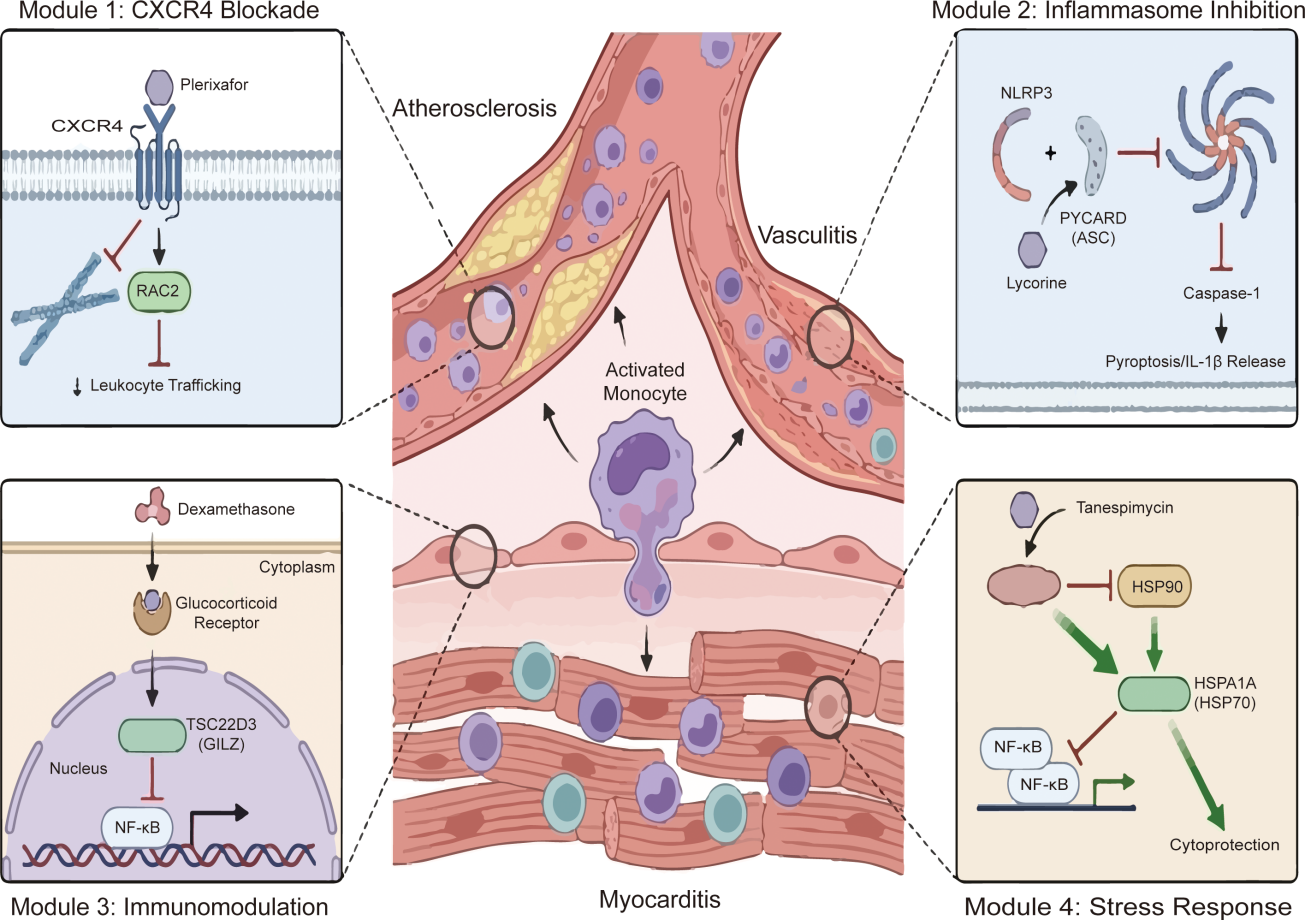
Therapeutic targeting of shared mechanistic modules across cardiovascular inflammatory diseases. The central schematic illustrates the convergence of atherosclerosis, vasculitis, and myocarditis mediated by activated monocytes. Four key molecular targets and their respective pharmacological modulators are highlighted in detailed insets. Module 1: CXCR4 Blockade. Plerixafor antagonizes the CXCR4 receptor, blocking downstream RAC2 signaling to inhibit leukocyte trafficking. Module 2: Inflammasome Inhibition. Lycorine interferes with the interaction between NLRP3 and PYCARD (ASC), preventing Caspase-1 activation, IL-1β release, and pyroptosis. Module 3: Immunomodulation. Dexamethasone binds to the glucocorticoid receptor in the cytoplasm to induce TSC22D3 (GILZ) expression, which enters the nucleus to repress the NF-κB signaling pathway. Module 4: Stress Response. Tanespimycin inhibits HSP90, triggering a compensatory upregulation of HSPA1A (HSP70). Elevated HSPA1A exerts cytoprotective effects by suppressing NF-κB activation. CXCR4, C-X-C chemokine receptor type 4; RAC2, Rac family small GTPase 2; NLRP3, NLR family pyrin domain containing 3; PYCARD, PYD and CARD domain containing; ASC, Apoptosis-associated speck-like protein containing a CARD; TSC22D3, TSC22 domain family member 3; GILZ, Glucocorticoid-induced leucine zipper; HSPA1A, Heat shock protein family A (Hsp70) member 1A; NF-κB, Nuclear factor kappa-light-chain-enhancer of activated B cells.

The CXCR4/CXCL12 axis exerts a context-dependent dual physiology (**Figure 5**). While acting as a central conduit for pathological infiltration, it is simultaneously indispensable for vascular homeostasis. Mechanistically, CXCR4 engages the DOCK-RAC2 pathway to orchestrate cytoskeletal rearrangement and endothelial penetration (178). Clinically, CXCR4 expression correlates with plaque vulnerability in AS (179) and drives the expansion of cytotoxic CD8+ T cells in viral myocarditis (124). Furthermore, chemokines direct the pathological localization of B cells within cardiovascular tissues, exacerbating the vascular inflammatory milieu (180). This pleiotropic axis mediates diverse cardiovascular processes, ranging from platelet-derived CXCL12-mediated inflammatory exacerbation to the recruitment of bone marrow progenitors for tissue repair (172). Conversely, this axis mediates tissue repair; MEK1/2 inhibitors have been shown to promote endothelial regeneration specifically via the miR-126-3p/CXCL12/CXCR4 cascade (181, 182), and CXCR4 signaling facilitates the homing of regenerative M2 macrophages (183). Crucially, moderate CXCR4 activation directly potentiates endothelial proliferation, indicating that preserving basal signaling is indispensable for vascular integrity (184). Given this complex landscape, pharmacological modulation requires precision. While the antagonist Plerixafor effectively blocks CXCR4-mediated infiltration (174), complete blockade must be carefully balanced against these homeostatic functions. To address this, refined strategies such as targeting CXCR4-MIF heterocomplexes with zedoarondiol offer a way to reduce monocyte adhesion while preserving essential signaling pathways (185, 186).

### 6.3 PYCARD and the NLRP3 inflammasome

PYCARD, which encodes the adaptor protein ASC, bridges metabolic stress signals to innate immunity (**Figure 5**). Within the NLRP3 inflammasome, ASC utilizes its PYD and CARD domains to recruit pro-caspase-1, triggering GSDMD-mediated pyroptosis and the release of proinflammatory cytokines, specifically IL-1β and IL-18 (100, 187). In AS, ASC acts as a sensor for lysosomal damage induced by cholesterol crystals (105) and is upregulated by PCSK9 (188). This hyperlipidemic stress activates the NLRP3/ASC/Caspase-1 axis, destabilizing plaques (189), while elevated ASC similarly correlates with severity in myocarditis (190). Beyond expression levels, activation is strictly governed by post-translational modifications. Structurally, differential ASC isoform expression dictates whether the inflammasome pathologically self-assembles or remains dormant under metabolic stress (170). A tyrosine phosphorylation switch functions as a critical checkpoint: while phosphorylated in the resting state, ASC must undergo dephosphorylation to permit oligomerization (191). Concurrently, USP25 promotes assembly by deubiquitinating NLRP3 to prevent its degradation (192). Therefore, strategies aimed at disrupting ASC assembly represent a critical intervention point. MCC950 directly blocks NLRP3-induced ASC oligomerization to suppress pyroptosis (105, 193), and existing agents like colchicine and the SGLT2 inhibitor canagliflozin have also been shown to downregulate ASC complexes (105, 194). Notably, recent studies highlight Lycorine as a potent agent that specifically targets the ASC PYD domain. By physically disrupting the NLRP3-ASC interaction, Lycorine serves as a robust strategy to arrest the inflammatory cascade (175), further validating the PYCARD axis as a druggable target.

### 6.4 TSC22D3 and immune tolerance

TSC22D3, also known as GILZ, functions as a constitutive molecular brake maintaining cardiovascular immune homeostasis(**Figure 5**). Physiologically induced by laminar shear stress, GILZ suppresses NF-κB translocation, thereby silencing adhesion molecules and chemokines (195). Functioning as a central immunomodulator, it drives reparative M2 macrophage polarization and promotes regulatory T cell differentiation to mitigate autoimmune tissue damage (171). Loss of this checkpoint due to altered glucocorticoid metabolism accelerates AS (196) and fibrosis (176, 197). Consequently, interventions capable of restoring GILZ activity are vital for re-establishing intrinsic tolerance. Dexamethasone and synthetic glucocorticoids remain the primary pharmacological agents for upregulating GILZ (198). By reinforcing this endogenous checkpoint, such treatments can effectively block systemic inflammatory cascades, although balancing efficacy with metabolic side effects remains a priority for the development of novel analogs.

### 6.5 HSPA1A and proteostatic stress

HSPA1A constitutes a core component of the proteostatic defense network, which is upregulated as an adaptive response to stress (**Figure 5**) (199). It functions as a cytoprotective shield by inhibiting the NF-κB pathway to dampen pro-inflammatory cytokines and block apoptosis (177). This mechanism underpins vascular repair, such as IL-28A-promoted endothelial angiogenesis (200). Pathologically, HSPA1A exerts dichotomous effects: while intracellularly cytoprotective, its extracellular release acts as a circulating biomarker correlating with systemic inflammation, aging, and classical vascular risk factors (173). Pharmacologically boosting this defense mechanism offers a promising therapeutic avenue. HSP90 inhibitors, such as Tanespimycin, exert therapeutic effects by inducing a compensatory upregulation of HSPA1A (177). This upregulation reinforces the anti-inflammatory shield. However, context is crucial, as excessive HSPA1A in autoimmune vasculitis may confer resistance to proteasome inhibitors (201), underscoring the need for precision dosing when targeting the heat shock response.

## 7 Cross-talk among AS, myocarditis and vasculitis

### 7.1 Common pathogenic factors

AS, myocarditis and vasculitis share pathogenic convergences involving oxidative stress, endothelial dysfunction, and epigenetic dysregulation. Mechanistically, the NLRP3 inflammasome acts as a universal sensor orchestrating the core inflammatory response (202). This cascade is modulated by a multidimensional neuroendocrine network, where sympathetic activation promotes inflammation, while parasympathetic signals and hormones including glucocorticoids, melatonin, and oxytocin exert compensatory immunoregulation (203).

### 7.2 The cardiovascular-immune axis

The relationship between vasculitis and AS involves a self-perpetuating causal cycle, wherein inflammation within atherosclerotic vessel walls is itself driven by innate and adaptive immune responses (204). Systemic cytokines including IL-1β, IL-6, and TNF, alongside direct vascular injury, increase adhesion molecule density to create a permissive environment for atherogenesis. Crucially, MPO bridges these pathologies by catalyzing intimal LDL oxidation to facilitate scavenger receptor-mediated uptake and foam cell formation (83, 138). This link is compounded by a “therapeutic paradox”: glucocorticoids used for vasculitis induce metabolic sequelae such as hypertension, hyperlipidemia, and hyperglycemia, which paradoxically exacerbate plaque progression (138). Therapeutically, targeting the GILZ hub resolves this paradox. GILZ mimetics selectively mediate glucocorticoid transrepression while bypassing the transactivation pathways responsible for metabolic dysregulation, thereby suppressing vasculitis and preventing accelerated atherogenesis.

Epigenetically, lncRNAs play a crucial role. MALAT1 deletion and MIR181A1HG upregulation promote endothelial inflammation (205), while MAP3K4 maintains barrier integrity (206). Furthermore, MIR181A1HG deletion in ECs significantly attenuates vascular inflammation (156), ultimately slowing the progression of AS. Signaling axes including acacetin-activated Nrf2 and the PTP4A1/USF1/A20 pathway further regulate endothelial function; PTP4A1 downregulation compromises the A20 brake, leading to unchecked inflammation (207–209). In the context of viral triggers, endothelial RIG-I initiates interferon responses (210), linking viral myocarditis to vasculitis, a continuum evident in KD models where IL-1 signaling accelerates AS (211–213). Furthermore, ICIs disrupt homeostasis, frequently precipitating myocarditis while accelerating AS (135, 214).

### 7.3 Therapeutic prospects and novel strategies

The shared molecular architecture supports unified therapeutic strategies. These interventions must be carefully stratified into approved therapies specifically for cardiovascular indications, repurposed investigational agents, and preclinical candidates, with close attention paid to their respective toxicity profiles.

Targeting the NLRP3 inflammasome and downstream cytokines represents a clinically validated frontier. The landmark Canakinumab Anti-inflammatory Thrombosis Outcome Study provided definitive proof-of-concept, demonstrating that neutralizing interleukin-1 beta significantly reduces cardiovascular events, albeit alongside an increased risk of fatal systemic infections (215). Colchicine, widely repurposed for cardiovascular risk reduction, is limited by a narrow therapeutic index, featuring gastrointestinal intolerance and severe synergistic myotoxicity when co-administered with statins due to cytochrome P450 3A4 interactions (216). Furthermore, the interleukin-1 receptor antagonist anakinra remains under investigational evaluation for cardiovascular indications, requiring stringent monitoring for severe neutropenia and opportunistic infections (217). In the preclinical arena, agents like tranilast restrict inflammasome assembly via ubiquitination, while natural phytochemicals offer supplementary inhibition; however, their clinical translation is severely hindered by poor systemic bioavailability and rapid metabolic clearance (218–220).

Modulating immune cell trafficking presents another promising therapeutic avenue. Plerixafor, an approved CXC motif chemokine receptor 4 antagonist, is currently under investigational use to block pathological leukocyte infiltration, though its broad systemic effects necessitate careful evaluation to prevent off-target immunological disruption (174). To overcome such systemic toxicities, preclinical platforms including biomimetic rapamycin-loaded leukosomes have been designed for targeted endothelial delivery. This localized approach specifically circumvents the profound immunosuppression and metabolic dysregulation classically associated with systemic rapamycin therapy (221).

For concurrent cardiovascular and immune emergencies, classical mainstays are frequently combined. High-dose glucocorticoids remain the first-line defense, yet prolonged use introduces a therapeutic paradox driven by severe metabolic toxicities and heightened infection susceptibility (222). Statins confer dual anti-inflammatory and endothelial benefits but carry dose-dependent risks of myopathy, rhabdomyolysis, and transaminase elevations (223). Additionally, metabolic regulators such as glucagon-like peptide 1 receptor agonists significantly reduce major adverse cardiovascular events, provided that gastrointestinal intolerances and rare pancreatic toxicities are carefully managed (224).

Beyond classical targets, preclinical interventions inhibiting the multifunctional kinase CK2 or the necrotic effector RIPK1 confer protection against viral and autoimmune injury (225, 226). Nevertheless, because these kinases are essential for fundamental cellular survival, systemic pharmacological inhibition risks lethal off-target tissue damage (225, 226). Finally, nucleic acid therapeutics present contrasting translational landscapes. Small interfering RNA agents targeting proprotein convertase subtilisin/kexin type 9 achieve sustained lipid lowering with a highly favorable safety profile. Conversely, investigational therapies aiming to modulate microRNAs or facilitate messenger RNA-driven endothelial repair face massive translational hurdles regarding *in vivo* delivery efficiency and immunogenicity (227). These precedents underscore the critical necessity of balancing target specificity with systemic safety in cardiovascular immunopharmacology.

## 8 Conclusion and future perspectives

In conclusion, despite diverse metabolic or autoimmune triggers, AS, myocarditis, and vasculitis converge on a shared immune landscape anchored by CXCR4, PYCARD, TSC22D3, and HSPA1A. Consequently, agents such as plerixafor, lycorine, dexamethasone, and tanespimycin emerge as promising cross-disease modulators. Current strategies, however, are constrained by insufficient stage specificity. Future research must therefore prioritize deciphering spatiotemporal immune dynamics to facilitate a shift from broad-spectrum immunosuppression to precision immunomodulation. Ultimately, harnessing emerging modalities like RNA therapeutics and cell-specific nanomedicines will enable a regulatory paradigm that resolves inflammation while preserving cardiovascular homeostasis.
